# Nanomaterials for Ion Battery Applications

**DOI:** 10.3390/nano12132293

**Published:** 2022-07-04

**Authors:** Jaehyun Hur

**Affiliations:** Department of Chemical and Biological Engineering, Gachon University, Seongnam 13120, Gyeonggi, Korea; jhhur@gachon.ac.kr; Tel.: +82-31-750-5593

Nanomaterials offer opportunities to improve battery performance in terms of energy density and electrochemical reaction kinetics owing to a significant increase in the effective surface area of electrodes and reduced ion diffusion pathways. Nevertheless, a large number of unwanted secondary reactions in nanomaterials, which lead to the rapid deterioration in prolonged cycling, are important issues to be overcome. Therefore, the judicious design of nanoarchitecture is one of the essential themes in battery research. The Special Issue of “Nanomaterials for Ion Battery Applications” of *Nanomaterials* covers the recent advancements in nanotechnologies and nanomaterials for various ion batteries including Li-ion batteries (LIBs), Li-O_2_ batteries, and multivalent aqueous batteries. Seeking facile, inexpensive, and scalable processes to synthesize new nanomaterials and nanoarchitectures into high-performance ion batteries is another important research topic in this Special Issue.

The development of new, high-performance anode materials for LIBs was intensively reported in this Special Issue. Nguyen et al. demonstrated various important roles of layered-structure transition-metal chalcogenide (TMC) materials for LIBs. Although MoS_2_ is one of the most widely studied TMC materials for LIB anodes, they showed new nanostructures and functions of MoS_2_. First, they demonstrated that few-layer MoS_2_ covered SnO_2_ nanoparticles (NPs) to achieve the highly reversible and high-capacity anode material for LIBs [1]. Few-layer 1T MoS_2_ nanosheets prepared using a conventional liquid chemical exfoliation method (lithium intercalation in butyllithium-dissolved hexane) was coated on the SnO_2_ NPs via layer-by-layer fashion at the air–water interface. The coverage of few-layer MoS_2_ on SnO_2_ contributed to the reduced volume expansion of active SnO_2_ NPs, which improved the reversibility of lithiation/delithiation. After optimizing the number of MoS_2_ layers, they found single-layer MoS_2_-coated SnO_2_ NPs exhibited good cycling performance. Second, few-layer 1T MoS_2_ was demonstrated as an active material for the high-performance LIB anode by the same group [2]. Because of the cumbersome restacking of few-layer MoS_2_ during the electrochemical reactions, they introduced Ag NPs in the MoS_2_-dispersed solution via a simple reduction of AgNO_3_ with NaBH_4_. The uniform distribution of Ag NPs (hundreds of nanometers) on few-layer MoS_2_ was facilitated by the 3-mercaptopropionic acid. The resulting Ag@MoS_2_ electrode exhibited good cycling performance (510 mAh g^−1^ after 100 cycles) and rate performance. Third, a WS_2_/W_2_C composite material was demonstrated as a great candidate TMC composite material for the LIB anode [3]. Although the flower-like WS_2_/W_2_C nanostructure possesses high surface-to-volume ratio, it showed unstable cycling behavior. The in situ incorporation of carbon nanotubes (CNTs) in WS_2_/W_2_C highly improved electrical conductivity without significant agglomeration of CNTs. The obtained performance of the WS_2_/W_2_C-CNT composite anode (650 mAh g^−1^ at 100 mA g^−1^ after 100 cycles) was better than those of most WS_2_-based anodes with various nanostructures. Metallic alloy is another high-potential anode for LIBs. Huy et al. reported C-decorated InSb alloy (InSb-C) as a potential anode material for LIBs [4]. InSb-C was synthesized via a ball-milling process simply using In, Sb, and C as reacting materials. The effects of the binder and buffering matrix on the InSb anode performance were systematically studied. Owing to the hydroxyl functional groups on InSb particles, the poly(acrylic acid) containing carboxylate groups showed better adhesion and stability as a binder than a conventional poly(vinylidene fluoride) binder. The addition of amorphous C in InSb enhanced mechanical stability and electrical conductivity. The InSb-C delivered a high reversible capacity (878 mAh g^−1^ at 100 mA g^−1^ after 140 cycles) and outstanding rate capability (capacity retention of 98% at 10 A g^−1^ relative to 0.1 A g^−1^). Semiconducting metal oxides (SMOs) are widely studied materials for high-performance LIB anodes owing to high theoretical capacity and safety. Among various SMOs, Bui et al. reviewed ZnO-based binary and ternary composites for LIB anodes [5]. ZnO possesses various benefits such as high theoretical capacity (978 mAh g^−1^), low-cost, environmental friendliness, high chemical and mechanical stability, and abundance. However, it generally shows a severe capacity fading during cycling due to the remarkable volume change (228%) and aggregation. This review gave an overview of the ZnO binary and ternary composites that overcame these problems via new designs of materials, structures, and processes, which provides useful guidance for the development of not only ZnO-based anode material, but also other SMO-based anode materials.

Aqueous rechargeable batteries are promising alternatives for LIBs because of safety and facile cell assembly. Among these, aqueous Zn-ion batteries (AZIBs) have emerged as a promising technology owing to high theoretical capacity (820 mAh g^−1^, 5854 mAh L^−1^), abundance, low-cost, and appropriate redox potential (−0.76 V vs. standard hydrogen electrode). However, the performance of current AZIBs is not satisfactory due to various issues in both the cathode and anode. Therefore, the development of new materials for the cathode and anode that can overcome such issues are of great importance in this research field. Kim et al. synthesized NH_4_V_4_O_10_ (NHVO) flower-like layered oxide via a simple and rapid microwave method to use as a high-performance cathode material for AZIBs [6]. Although vanadium-based cathodes have been intensively studied, they have been mainly synthesized using the hydrothermal process. The microwave synthesis of NHVO significantly reduced a synthesis time without sacrificing the performance. The NHVO cathode delivered 277 mAh g^−1^ with 75% capacity retention after 150 cycles at 0.25 A g^−1^ and showed exceptional high coulombic efficiencies. Meanwhile, the utilization of TMCs as promising cathode materials in AZIBs, as well as other aqueous multivalent metal-ion batteries, was extensively reviewed by Huy et al. [7]. The interlayer spacing of various TMCs is the key aspect of cathode materials to be successfully used in various multivalent ion-based aqueous batteries. Various strategies including (i) intercalation modification, (ii) defect modification, (iii) hybridization, and (iv) phase modification were explained and future research directions were discussed in this review. On the anode side, one of the most important hurdles is the control of random Zn dendrite growth and hydrogen evolution on Zn metal. Liu et al. proposed that the Cu–Zn alloy can significantly improve the Zn metal performance [8]. The Cu–Zn alloy was fabricated simply by dropping 0.1 M of CuSO_4_ aqueous solution (100 μL) on Zn foil and allowed to sit for 3 min, followed by washing and drying. A spontaneous replacement reaction changed the Zn foil color from gray to black, indicating the formation of the Cu–Zn alloy. Benefitting from reduced overpotential, the Cu–Zn alloy guided the uniform Zn nucleation, resulting in the remarkable improvement in cycling life of symmetric cells. Furthermore, when coupled with the V_2_O_5_-PEDOT cathode, the full aqueous battery exhibited stable and reversible cycling behavior over 1000 cycles. The general challenges and strategies of Zn metal anodes in mild aqueous electrolytes are extensively summarized by Huy et al. [9]. The promising strategies were categorized to be (i) Zn metal shielding, (ii) control of Zn nucleation and Zn-ion flux redistribution, and (iii) establishment of uniform electric field. This review presented design guidelines for the development of high-performance AZIBs.

Li-O_2_ batteries have drawn significant attention as next-generation energy storage with high energy density (10 times higher than conventional LIBs), low-cost, and green technology. However, the practical viability has been challenging due to sluggish O_2_ reaction kinetics. Karuppiah et al. proposed the La_0.8_Ce_0.2_Fe_0.5_Mn_0.5_O_3_ perovskite oxide/graphene nanosheet composite (LCFM8255/GNS) as a potential cathode for Li-O_2_ batteries, which can resolve such problems [10]. Mesoporous LCFM8255 (high crystallinity and specific surface area)-embedded GNS boosted the electrochemical kinetics owing to its structural benefits. In addition, a high capacity (8475 mAh g^−1^) and cycle stability were achieved at the current density of 100 mA g^−1^.

A gel polymer electrolyte (GPE) is one of the solid electrolytes that can overcome the safety issues in organic solvent-based liquid electrolytes. In GPEs, the introduction of inorganic fillers is an effective strategy to achieve high ionic conductance and strong interfacial contact with an electrode. Huy et al. provided an overview of recently reported studies regarding the detailed functions of inorganic fillers including TiO_2_, Al_2_O_3_, SiO_2_, ZrO_2_, CeO_2_, and BaTiO_3_ added in GPEs for various batteries (Li, Na, Mg, and Zn-ion batteries) [11].

To summarize, this Special Issue covers the progress in various battery systems. The recent studies conducted by many dedicated researchers highlight the importance of innovative nanostructures and new functional materials. Electrochemical performance is highly correlated with these, which can open up new opportunities for battery research.
